# Coupling Microplate-Based Antibacterial Assay with Liquid Chromatography for High-Resolution Growth Inhibition Profiling of Crude Extracts: Validation and Proof-of-Concept Study with *Staphylococcus aureus*

**DOI:** 10.3390/molecules26061550

**Published:** 2021-03-11

**Authors:** Hamidreza Ardalani, Syariful Anam, Kresten J. K. Kromphardt, Dan Staerk, Kenneth T. Kongstad

**Affiliations:** 1 Department of Drug Design and Pharmacology, Faculty of Health and Medical Sciences, University of Copenhagen, Universitetsparken 2, DK-2100 Copenhagen, Denmark; hamidreza.ardalani@agro.au.dk (H.A.); syariful.anam@untad.ac.id (S.A.); ds@sund.ku.dk (D.S.); 2 Department of Pharmacy, Faculty of Mathematics and Sciences, Tadulako University, Jalan Soekarno Hatta Km. 9, Palu 94118, Central Sulawesi, Indonesia; 3 Department of Biotechnology and Biomedicine, Technical University of Denmark, Søltofts Plads, Building 223, 2800 Kongens Lyngby, Denmark; krjko@dtu.dk

**Keywords:** dye-free microplate assay, antibacterial, high-resolution antibacterial profiling, natural products, *Staphylococcus aureus*

## Abstract

With the identification of novel antibiotics from nature being pivotal in the fight against human pathogenic bacteria, there is an urgent need for effective methodologies for expedited screening of crude extracts. Here we report the development and validation of a simple and dye-free antimicrobial assay in 96-well microplate format, for both determination of IC_50_ values and high-resolution inhibition profiling to allow pin-pointing of bioactive constituents directly from crude extracts. While commonly used antimicrobial assays visualize cell viability using dyes, the developed and validated assay conveniently uses OD_600_ measurements directly on the fermentation broth. The assay was validated with an investigation of the inhibitory activity of DMSO against *Staphylococcus aureus*, temperature robustness, interference by coloured crude extracts as well as inter-day reproducibility. The potential for high-resolution *S. aureus* growth inhibition profiling was evaluated on a crude extract of an inactive *Alternaria* sp., spiked with ciprofloxacin.

## 1. Introduction

The extensive use of antibiotics in agriculture, animal production, and human health care has resulted in emerging problems with multi-resistant bacteria, and consequently a large increase in mortality rates, with an estimated annual cost in the OECD and EU countries of more than USD 3.5 billion, 60,000 deaths, and 700 million extra hospital days [1]. To be able to respond to the bacterial challenge posed by the ever-emerging multi-resistance, now even covering the last-resort compound class of colistins [2], the development of novel antibiotics with a new mode-of-action is crucial. 

Nature has been and still is, an important source for discovering novel drugs with new modes-of-action, as reviewed by Newman and Cragg [3]. In the area of antibacterial agents, natural products and natural product-derived compounds account for 73% of the approved agents between 1981 and 2014 [3]. Importantly, as emphasized by Silver [4], no new antibacterial compound classes have been identified since 1987, with the diarylquinoline bedaquiline, marketed in 2012, as the only exception. Thus, with the continued emergence of multi-resistant bacteria, there is a need to investigate new biological material as sources for antibacterial drug leads. Historically, natural products derived from microorganisms, including endophytic fungi [5,6,7,8], have been promising sources of bioactive natural products. Since the discovery of paclitaxel from the endophytic fungus *Taxomyces andreanae,* many new bioactive natural products from endophytic fungi have been reported [9]. Several approaches are used for discovering bioactive constituents from crude extracts of bacterial and fungal cultures. One of the most efficient is to combine analytical-scale microfractionation with a microplate-based assay to perform a so-called high-resolution inhibition profiling. This has been done for various targets, such as the α-glucosidase and PTP1B enzymes [10], fungal plasma membrane H^+^-ATPase [11], and snake venom enzymes [12]. Despite high-resolution inhibition profiling combined with hyphenated high-performance liquid chromatography—photodiode array detection—high-resolution mass spectrometry—solid-phase extraction—nuclear magnetic resonance (HPLC-PDA-HRMS-SPE-NMR) [13] is currently perceived as state-of-the-art for natural product drug discovery [14,15], no such method, however, has been reported for the discovery of antibacterial compounds from nature. One of the simplest screening techniques for antibacterial activity of natural products or extracts is the disk diffusion assay [16]. This is, however, limited by low sensitivity and inability to determine precise IC_50_ values of the crude extracts [17] and not suitable for high-resolution purposes. Minimum inhibitory concentration (MIC) of crude extracts and purified compounds against bacteria are commonly determined in microplate-based assays, potentially being suitable for high-resolution bioactivity profiling. These microplate-based assays use various dyes such as tetrazolium salts [18] and resazurin [19] as indicators for cell viability. The most widely used tetrazolium salt, MTT (3-(4,5-dimethylthiazol-2-yl)-2,5-diphenyltetrazolium bromide), is intra-cellularly converted from a colourless compound into a water-insoluble, blue-coloured formazan. While MIC determination can be done by visually observing the blue colour-change, photometric MTT assays require additional cell lysis and formazan-dissolving steps prior to analysis [20]. Although MIC-determination is a commonly used method, it is intrinsically qualitative and unsuitable for statistic calculations [21]. IC_50_ determination is a quantitative method that allows calculation of standard deviation. ’In order to study bacterial strains’ subtle differences in susceptibility to antibacterial compounds, IC_50_-determination is considered more suitable and useful than MIC-determination [21]. Additionally, dye-based microplate assays require several pipetting steps for exchanging culture media with dye and additional incubation time for conversion of the tetrazolium salt. To expedite antibacterial drug discovery from nature, the aim of this work was to develop and validate a simple, dye-free microplate assay suitable for high-resolution inhibition profiling of both major and minor constituents directly from crude extracts. The commonly used Gram-positive bacterium *Staphylococcus aureus* was chosen as a test organism for this proof-of-concept study using optical density (OD) measurements for profiling purposes.

## 2. Results and Discussion

This work describes the validation of OD measurements for evaluation of antimicrobial activity, which allows quantitative high-throughput screening of crude natural product extracts and high-resolution *S. aureus* growth inhibition profiling. The drawbacks of the commonly used dye-based microplate assays, which include issues with solubility, extensive pipetting and sensitivity to temperature [20], led us to choose OD_600_ as a simple, quantitative measure of the turbidity, corresponding to *S. aureus* growth. As microplate-based high-resolution inhibition profiling is based on microfractionation and single-well measurements, the assay must be validated, and experimental variations investigated and preferentially eliminated. Thus, different experimental factors potentially affecting the observed inhibition including temperature, solvent ratio, and photometric condition were investigated by performing IC_50_ determinations at varying conditions as detailed below.

### 2.1. Temperature Robustness of the Assay

**Figure 1:**
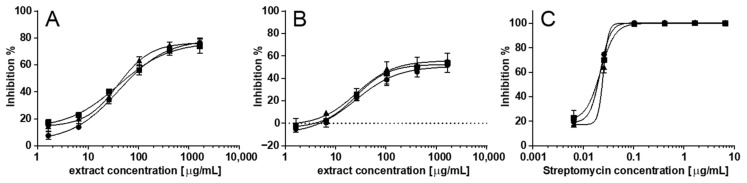
IC_50_ curves of crude extracts of the endophytic fungi *A. montevidensis* (**A**) and *T. asperellum* (**B**), and the reference compound, streptomycin (**C**), at 34 °C (circle, ●), 37 °C (square, ■) and 40 °C (triangle, ▲). Measurements were performed in triplicates, representing inter-day reproducibility, and plotted as mean inhibition (%) ± standard deviations against extract concentration (µg/mL).

The developed microplate-based bacterial growth inhibition assay uses an incubation temperature of 37 °C, which is common for studies on *S. aureus* [18,22,23]. To ensure that the developed assay do not share the same drawbacks as the dye-based cell-viability assays in terms of sensitivity to temperature changes, affecting the reaction rate, the robustness to temperature fluctuations was investigated. Streptomycin and crude extracts of the antibacterial endophytic fungi, *Aspergillus montevidensis* and *Trichoderma asperellum*, were analysed at 34, 37 and 40 °C in triplicates to investigate how the determined IC_50_ values were affected. The two endophytic fungi *A. montevidensis* and *T. asperellum* showed reproducible IC_50_ values at 37 °C of 39.91 ± 1.36 µg/mL and 25.94 ± 0.62 µg/mL, respectively (Table 1 and Figure 1). It was furthermore concluded that there was no significant difference in IC_50_ values due to temperature variations according to a one-way ANOVA analysis (*p* value > 0.05). 

### 2.2. Staphylococcus aureus Growth Inhibitory Effect of DMSO 

**Figure 2:**
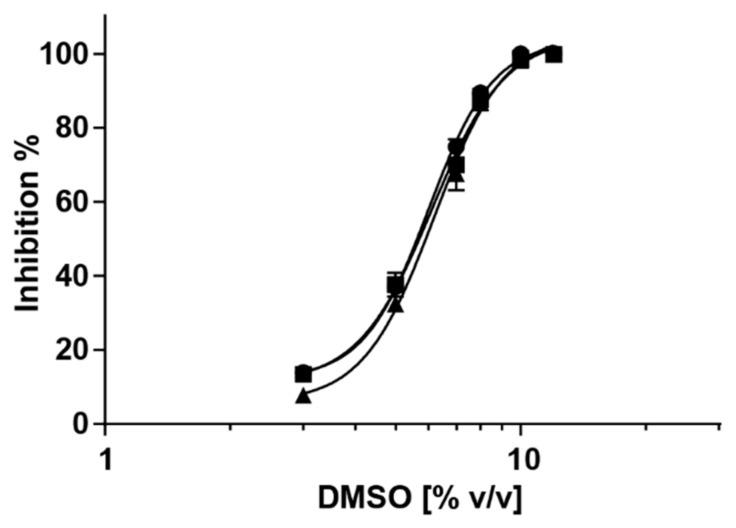
Inhibition curves of DMSO against *S. aureus* at 34 °C (circle, ●), 37 °C (square, ■) and 40 °C (triangle, ▲). Measurements performed in triplicates on individual days and plotted as mean inhibition (%) ± standard deviations against DMSO (% *v*/*v*).

DMSO is often used as solvent for dissolving metabolites prior to being added to aqueous assays. This is particularly necessary when screening crude extracts due to the chemical complexity and broad range of polarities, complicating the ability to solubilise these in aqueous broth. While high concentrations of DMSO is attractive concerning solubility, it has a detrimental effect on cell viability at high concentrations [24]. Thus, the optimal final concentration of DMSO used in the assay and the effect on *S. aureus* viability was investigated. Using varying percentages of DMSO as the test compound and a blank control with only broth as the reference solution, IC_50_ curves at 34, 37 and 40 °C were constructed (Figure 2). It was found that the IC_50_ of DMSO on *S. aureus* is 6.00 ± 0.07% *v*/*v*, which is in accordance with a previous study of the effect of DMSO on *S. aureus* viability [25]. Based on these findings, it was decided to use a DMSO concentration of 5% *v/v* in the remaining experiments as a compromise between *S. aureus* inhibition and the ability to sufficiently dissolve the crude extracts.

### 2.3. Evaluation of the Interference by Coloured Crude Extracts 

**Figure 3:**
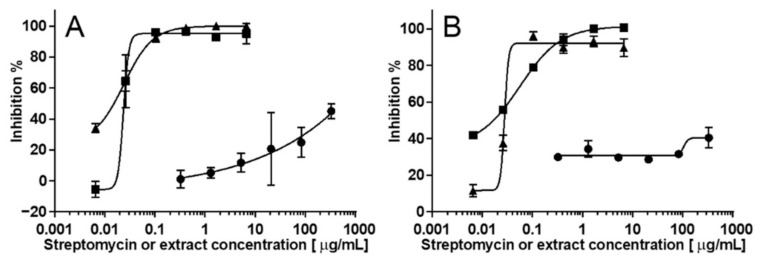
Inhibition curves of streptomycin (square, ■), spiked (triangle, ▲) and un-spiked (circle, ●) crude extracts of the endophytic fungus *Alternaria* sp. (**A**) and the leaves of *J. oxycedrus* (**B**) against *S. aureus* at 37 °C. Measurements performed in triplicates and plotted as mean inhibition (%) ± standard deviations against extract or streptomycin concentration (µg/mL).

Crude extracts are often coloured, which, when conducting photometric assays, could potentially have an adverse effect on the validity of the results. Crude coloured extracts of a plant and a filamentous endophytic fungus, *Juniperus oxycedrus* and *Alternaria* sp., respectively, showed no significant inhibitory activities towards *S. aureus* at the tested concentrations (Figure 3 and Table 1). To investigate if coloured constituents in the crude extract caused interference with the assay, both extracts were spiked with streptomycin, corresponding to the concentration range of the reference compound. With an IC_50_ value of the spiked crude extract of *Alternaria* sp. of 0.02 µg/mL, identical to that of streptomycin, it was concluded that the yellow color of the extract did not give rise to interference. However, the extract of *J. oxycedrus* did have an effect on the measured IC_50_ value (0.05 µg/mL), underestimating the potency of the spiked extract, compared to the expected 0.02 µg/mL. This is speculated to be attributed to the presence of chlorophyll as observed by characteristic UV spectra of hydrophobic constituents in the extract. The green color of the chlorophyll could affect the measured absorbance at 600 nm. However, as chlorophyll can form micelles and thereby increase the turbidity of the solution, we speculate that this could be a likely reason for the increase in the observed IC_50_ value. An approach to alleviating the interference by chlorophyll and other apolar micelle-forming constituents would be to do solvent partitioning between 90% methanol and low-boiling petroleum ether. However, with high-resolution inhibition profiling in mind, where analytes are separated by HPLC, we found the small interference to be acceptable for routine screening of extracts.

### 2.4. High-Resolution Staphylococcus aureus Growth Inhibition Profiling 

**Figure 4:**
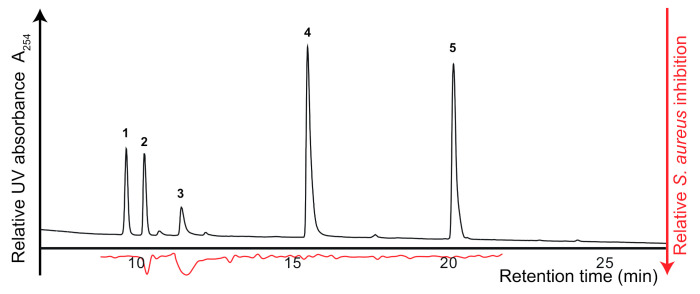
HPLC chromatogram at 254 nm of a test mixture of *S. aureus* inhibitors and inactive compounds, separated on a C_18_ column. The corresponding high-resolution *S. aureus* growth inhibition profile is shown below in red, pinpointing compounds **2** and **3** as *S. aureus* growth inhibitors.

For initial evaluation of the developed *S. aureus* growth inhibition assay for high-resolution growth inhibition profiling, an artificial mixture of known *S. aureus* inhibitors (ampicillin (**2**) and tetracycline (**3**)) and compounds with no known *S. aureus* inhibitory activity (metronidazole (**1**), myricetin (**4**), and podophyllotoxin (**5**)) was prepared. Each of the five compounds were mixed in methanol to a concentration of 60 μg/mL and separated by analytical-scale HPLC. The eluate from 9 to 21 min was collected into 88 wells of a 96-well microplate, leaving eight wells for blank samples, which led to a resolution of 4.4 data points per min. After evaporation of the HPLC eluate, the microfractionated extract was subjected to *S. aureus* growth inhibition assaying. The results, expressed as percent inhibition, was plotted at the corresponding retention times to provide high-resolution growth inhibition profiles (Figure 4), identifying both ampicillin (**2**) and tetracycline (**3**) as having inhibitory activities. 

**Figure 5:**
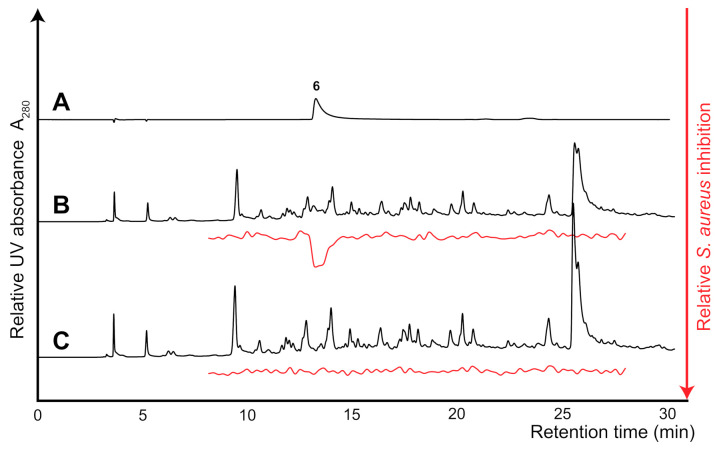
Comparison of HPLC chromatograms at 280 nm of ciprofloxacin (**A**), crude extract of *Alternaria* sp. (**C**) and the *Alternaria* sp. extract spiked with ciprofloxacin (**B**), separated on a C_18_ column. Corresponding high-resolution *S. aureus* growth inhibition profiles are shown below in red highlighting the *S. aureus* growth inhibition of ciprofloxacin.

With confirmation that the high-resolution inhibition profiling could pin-point the active constituents of the artificial test mixture (Figure 4), the inactive extract of the *Alternaria* sp. was spiked with the *S. aureus* inhibitor ciprofloxacin and both the neat and spiked extract were microfractionated for subsequent growth inhibition profiling. The eluates from 8 to 20 min were fractionated into 88 wells of 96-well microplates (4.4 data points per min) and subsequently evaporated before being subjected to growth inhibition assaying (Figure 5B,C). For reference, pure ciprofloxacin (**6**) was also analyzed (Figure 5A) As expected, no peaks were observed in the high-resolution inhibition profile of the crude *Alternaria* sp. extract (Figure 5C). However, when spiked with ciprofloxacin, a peak was observed in the high-resolution inhibition profile (Figure 5B) at the same retention time as observed in the HPLC chromatogram of ciprofloxacin (Figure 5A). This confirmed that the developed assay can effectively pinpoint natural products with *S. aureus* growth inhibition directly from crude natural product extracts and thus greatly expedite future antibiotic drug discovery.

**Figure 6:**
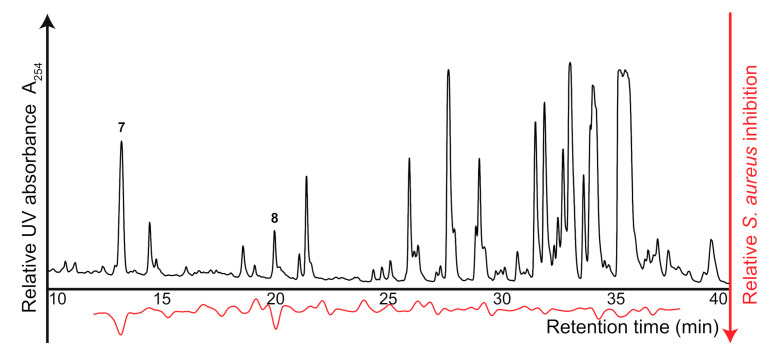
HPLC chromatogram at 254 nm of the crude extract of *A. montevidensis*, separated on a C_18_ column. The corresponding high-resolution *S. aureus* growth inhibition profile is shown below in red, pinpointing compounds **7** and **8** as *S. aureus* growth inhibitors.

The crude extract of *A. montevidensis* was subsequently fractionated into 88 wells (12 to 38 min, corresponding to 3.4 data points per min) and profiled for S. aureus inhibitory constituents. Notable inhibitions were observed at 13.3 min (**7**) and 20.1 min (**8**) (Figure 6). Dereplication based on HPLC-PDA-HRMS analysis of the crude extract suggested compound **7** as a di-hydroxylated benzoic acid (*m*/*z* 155.0343, corresponding to C_7_H_7_O_4_^+^, ΔM −2.6 ppm), which could be tentatively identified as 2,3-dihydroxybenzoic acid, previously isolated from A. fumigatus [26], due to the observed UV maxima (245 nm and 315 nm). Compound **8** showed a pseudomolecular ion of *m*/*z* 324.1696, corresponding to C_19_H_22_N_3_O_2_^+^ (ΔM −1.1 ppm) which led to a tentative identification as (+/−)-neoechinulin A which has previously been reported from A. variecolor [27]. 2,3-dihydroxybenzoic acid have previously been shown to possess *S. aureus* growth inhibitory activity [28] and should thus not be further investigated. (−)-Neoechinulin A, on the other hand, has been reported as inactive against *S. aureus* [29] while a hydroxylated analogue exhibited potent inhibitory activities [30]. Obtaining this knowledge directly from the crude extract allows for well-informed decision-making and emphasizes the potential of high-resolution growth inhibition profiling for expediting discovery of novel antibiotics from nature.

## 3. Materials and Methods

### 3.1. Chemicals

Mueller-Hinton Broth (MHB), Muller-Hinton Agar (MHA), streptomycin, ciprofloxacin, ampicillin, tetracycline, metronidazole, myricetin, podophyllotoxin, dimethyl sulfoxide (DMSO ≥ 99.5%), and 3-(4,5-dimethylthiazol-2-yl)-2,5-diphenyltetrazolium bromide (≥ 98%) were purchased from Sigma Aldrich (St. Louis, MO, USA). Potato dextrose agar (PDA), methanol, dichloromethane, HPLC-grade acetonitrile, and ethyl acetate were purchased from VWR (Fontenay-sous-Bois, France). Formic acid (FA) was purchased from Merck (Darmstadt, Germany). Water was purified by 0.22 µM membrane filtration and deionization by using a Barnstead Nanopure system from Thermo Scientific (Waltham, MA, USA) or a Milli-Q Plus system (Millipore, Billerica, MA, USA), while the DNeasy UltraClean Microbial kit was purchased from Qiagen (Hilden, Germany) and the GFX PCR DNA and Gel Band Purification Kit were purchased from GE Healthcare (Chicago, IL, USA).

### 3.2. Plant Collection and Extraction

The leaves of *Juniperus communis* and *J. oxycedrus* were collected from Alborz and Ardebil, respectively, Iran and identified by Dr Hossein Hashemi, Islamic Azad University, Science and Research Branch (Tehran, Iran). Leaves of *J. oxycedrus* (20 g) were dried at room temperature for 7 days and extracted with 500 mL methanol-ethyl acetate (1:1) by ultrasonication for 1 h. The extract was filtered through Whatman filter paper and concentrated on a rotary evaporator to yield 1.3 g crude extract.

### 3.3. Isolation of the Fungal Endophytes

Leaves of *J. communis* and *J. oxycedrus* were cut into 5 mm pieces, no longer than 48 h after collection, before being thoroughly washed in distilled water, followed by submersion in 70% ethanol for 1 min, 5% sodium hypochlorite for 2 min and thoroughly rinsed in sterile water 3 times. The surface sterilised plant tissues were transferred to PDA-filled Petri dishes and incubated at 25 °C for 12 days. Individual fungal species were transferred to new PDA-filled Petri dishes. Their micromorphology was investigated under a light microscope (1000× magnification), using aniline blue for staining. The fungal endophytes *Trichoderma asperellum* (ILF-006) from *J. communis*, *Aspergillus montevidensis* (ILF-009) and an unknown *Alternaria* sp. (ILF-013), both from *J. oxycedrus*, were identified through using amplicon-sequencing of key genes, given numerical identification numbers and deposited in the Department of Drug Design and Pharmacology fungal collection held in a −80 °C temperature-surveyed freezer at the University of Copenhagen. 

### 3.4. Identification of Fungi

Identification of the *Alternaria* sp. was done by extracting genomic DNA from mycelium of a seven-day-old culture growing on YES-filled Petri dish. A mycelial scrape was transferred to a FastPrep tube and 500 μL of breaking buffer with lithium acetate (2% triton x-100, 1% SDS, 100 mM NaCl, 10 mM Tris HCl, 1 mM EDTA, 200 mM LiAc), and 200 μL acid-washed glass beads (0.75–1.00 mm) were added. The FastPrep tube was transferred to a Thermo Savant FastPrep FP120 Cell Disruptor (Thermo Savant BIO101, Qbiogene, Cedex, France), and run for 40 s at speed 4. The tube was then transferred to a table centrifuge and centrifuged at 10,000× *g* for 30 s and 150 μL of the supernatant was transferred to an Eppendorf tube. To this, 15 μL 5 M NaCl and 400 μL ice-cold 96% ethanol was added, and the tube was spun in a table centrifuge at 10,000× *g* for 3 min. The supernatant was removed, and the tube was dried on a heating block at 50 °C. After heating, 200 μL of sterile MilliQ water was added to redissolve the gDNA to be used for PCR.

PCR amplification of the following marker sequences was done to identify the *Alternaria* sp.: The ITS rDNA was PCR amplified with the ITS5 and ITS4 primers [31], part of the glyceraldehyde-3-phosphate dehydrogenase locus (GPD) was amplified using the primers gpd1 and gpd2 [32], and the *Alternaria* allergen gene Alt a1 was amplified by primers Alt-for and Alt-rev [33]. Each 50 µL reaction mixture included 1U PfuX7 DNA polymerase [34], CXL buffer (20 mM Tris/HCl, 10 mM KCl, 6 mM (NH_4_)_2_SO_4_, 2 mM MgSO_4_, 0.1 mg/mL BSA and 0.1% Triton-X [34], 0.4 µM of each primer, 200 µM dNTPs, 3% *v/v* DMSO, 3% *v/v* MgCl_2_, ca. 10 ng gDNA and MilliQ water to 50 µL. The PCR reaction consisted of 98 °C for 3 min followed by 15 cycles of 98 °C for 30 s, touchdown annealing of 60–45 °C (−1 °C/cycle) for 30 s for ITS reactions, and 65–50 °C (−1 °C/cycle) for 30 s for Alt a1 and gpd reactions, followed by elongation at 72 °C for 1 min. The 15 cycles were followed by 20 cycles of 98 °C 30 s, annealing at 55 °C for 30 s for ITS reactions and 60 °C for 30 s for Alt a1 and gpd reactions, followed by elongation at 72 °C for 1 min. After the second round of cycles, a final elongation of 3 min at 72 °C was performed. The resulting DNA fragments were purified using the Illustra GFX PCR DNA and Gel Band Purification Kit following the manufacturer’s instructions and Sanger sequenced by Eurofins Genomics Sequencing GmbH (Köln, Germany). The ITS fragment was sequenced using the ITS5 and ITS4 primers, the gpd fragment was sequenced using the gpd1 and gpd2 primers and the Alt a1 fragment was sequenced using the Alt-for and Alt-rev primers. The obtained sequences were trimmed and assembled to consensus sequences using CLC Main Workbench (Qiagen).

In CLC Main workbench the resulting consensus sequences were put into a multilocus phylogeny with the sequences presented by Runa and co-workers [35]. The phylogeny (data not shown) placed the *Alternaria* sp. isolate in the *Ulocladium* I clade in close relation to *A. obovoidea*, *A. botrytis*, *A. cucurbitae*, and *A. terricola*. It is therefore likely that ILF-013 is a new species of endophytic *Alternaria*.

Extraction of gDNA from *A. montevidensis* and *T. asperellum* was done by using the Qiagen DNeasy UltraClean Microbial kit following the manufacturer’s instructions. The ITS rDNA region was PCR amplified by using ITS5 and ITS4 primers [31] as well as part of the BenA gene β-tubulin using the primers Bt2a and Bt2b [36]. Each 50 µL reaction mixture included 1U PfuX7 DNA polymerase [34], CXL buffer (20 mM Tris/HCl, 10 mM KCl, 6 mM (NH_4_)_2_SO_4_, 2 mM MgSO_4_, 0.1 mg/mL BSA and 0.1% Triton-X [34], 0.4 μM of each primer, 200 μM dNTPs, 3% *v/v* DMSO, 3% *v/v* MgCl_2_, ca. 10 ng gDNA and MilliQ water to 50μL. The PCR reaction consisted of 98°C for 3 min followed by 15 cycles of 98 °C for 30 s, touchdown annealing of 60–45 °C (−1 °C/cycle) for 30 s for ITS reactions and 65–50 °C (−1 °C/cycle) for 30 s for β-tubulin reactions, followed by elongation at 72 °C for 1 min. The 15 cycles were followed by 20 cycles of 98 °C 30 s, annealing at 53 °C for 30 s for ITS reactions and 63 °C for 30 s for β-tubulin reactions, followed by elongation at 72 °C for 1 min. After the second round of cycles, a final elongation of 3 min at 72 °C was done. The resulting DNA fragments were purified using the Illustra GFX PCR DNA and Gel Band Purification Kit and Sanger sequenced by Eurofins Genomics Sequencing GmbH. The ITS fragments were sequenced using the ITS5 and ITS4 primers, while β-tubulin fragments were sequenced using the Bt2a and Bt2b primers. The obtained sequences were trimmed and assembled to consensus sequences using CLC Main Workbench. The resulting consensus sequences were used for BLAST-n searches (NCBI, Bethesda, MD, USA) against the GenBank NR database. Accession numbers for included sequences are given in Supplementary Materials Tables S1–S3. 

### 3.5. Cultivation and Extraction of Endophytic Fungi

The endophytic fungi were three-point inoculated on 15 PDA-filled Petri dishes and incubated for 10 days at 25 °C. The colonies (including 5 mm of the surrounding agar) were extracted with 500 mL methanol-dichloromethane-ethyl acetate (1:2:3) containing 1% (*v*/*v*) formic acid by ultrasonication for 1 h. The extracts were filtered through Whatman filter paper and concentrated on a rotary evaporator to yield 170, 190, and 280 mg crude extract of *T. asperellum*, *A. montevidensis*, and *Alternaria* sp., respectively.

### 3.6. Bacteria Suspension

The bacteria suspension of *S. aureus* (ATCC 6538) was prepared from 10 single colonies of 24 h old bacteria grown on MHA. The colonies were transferred to 2 mL sterile water in 10 mL tubes followed by absorbance measurement at 600 nm (OD_600_) using a Shimadzu UV-1800 spectrophotometer (Shimadzu, Kyoto, Japan). Sterile water was added to a final OD_600_ of 0.236, corresponding to McFarland 0.67. The bacteria suspension was diluted 100-fold in MHB for a final suspension of 1 × 10^6^ CFU/mL [37].

### 3.7. Sample Preparation 

All fungal and plant extracts as well as the reference compound, streptomycin, were dissolved in 15% DMSO by ultrasonication and sequentially diluted four-fold with 15% DMSO in individual 2 mL vials to obtain a dilution series of 5.0–0.004 mg/mL, 1.0–0.001 mg/mL and 0.02–0.00002 mg/mL, respectively. 

### 3.8. Assay Development

The *S. aureus* inhibition assay was performed in 96-well microplates using a final volume of 225 µL. To all wells were added 75 µL MHB as well as 75 µL 15% DMSO (leading to a final well concentration of 5% DMSO), containing the crude extract, the reference compound streptomycin or no additional compounds considered the blank control. Furthermore, bacterial growth control wells consisted of 75 µL MHB and 75 µL sterile water while the sterile control wells contained 225 µL MHB. All wells were prepared in triplicates and the mean value was used for calculations. The assay was started by adding 75 µL of the 1 × 10^6^ CFU/mL *S. aureus* stock solution (except for the sterile control wells) and subsequently mixing on a Heidolph Titramax 100 plate shaker (Heidolph, Schwabach, Germany) for 1 min at 1000 rpm. The OD_600_ was measured immediately after mixing (T1), using a Versamax microplate reader (Molecular Devices, San Jose, CA, USA). After an additional 20 s mixing the covered microplate was incubated for 20 h at 37 °C followed by a second OD_600_ measurement (T2) and the percentage inhibition was calculated in Microsoft Excel 2010 using the following equation:

(1) $$\mathrm{Percentage}\;\mathrm{inhibition}=\;\frac{\mathrm{OD}600_{\mathrm{Blank}}\;–\;\left(\mathrm{OD}600_{T2}\;–\;\mathrm{OD}600_{T1}\right)}{\mathrm{OD}600_{\mathrm{Blank}}}\;\times 100\;\%$$

where OD_600 Blank_ is the absorbance of the blank control wells (T2–T1), OD_600 T2_ is the absorbance of wells after 24 h of incubation, and OD_600 T1_ is the absorbance of wells right before incubation.

IC_50_ curves were constructed in GraphPad Prism 7.00 (GraphPad Software Inc., San Diego, CA, USA) using the following four-parameter equation:

(2) $$F\left(x\right)=y_{min}+\frac{y_{\max\;-\;}y_{min}}{1+\;\left(\frac{x}{{\mathrm{IC}}_{50}}\right)}$$

where *y_min_* is the background, *y_max_* − *y_min_* is the *y*-range, and *x* is the concentration of the extract. The optimal DMSO concentration was evaluated together with the temperature robustness of the assay using varying conditions as described in detail below. Inter-day reproducibility was investigated through testing crude extracts of the endophytic fungi *A. montevidensis* and *T. asperellum* and the reference, streptomycin, on three sequential days.

### 3.9. Effect of DMSO 

To evaluate the effects of the DMSO concentration on the bacterial growth, a bioassay was performed using varying concentrations of DMSO as the sample solution, ranging from 3–12% *v*/*v*. The assay was performed according to the protocol described above with the only exception that the blank control was only added sterile water, which allowed determination of the IC_50_ value of DMSO. 

### 3.10. Validation by Spiking Inactive Extracts

The effect of coloured, crude extracts was investigated by spiking inactive fungal and plant extract with the reference compound, streptomycin. Extracts of the endophyte *Alternaria* sp. and leaves of *J. oxycedrus*, which showed no antibacterial activity against *S. aureus*, were dissolved in 15% DMSO and tested according to the protocol described above. In addition, the extracts were spiked with streptomycin in concentrations corresponding to the dilution series of streptomycin alone.

### 3.11. High-Resolution Staphylococcus aureus Growth Inhibition Profiling 

The chromatographic separations were performed on Agilent 1200 series HPLC (Agilent, Santa Clara, CA, USA), consisting of a G1311A quaternary pump, a G1322A degasser, a G1316A thermostatted column compartment, a G1315C photodiode-array detector, a G1367C high-performance auto-sampler, and a G1364C fraction collector, all controlled by Agilent ChemStation ver. B.03.02 software and equipped with a reversed-phase Luna C18(2) column, 150 × 4.6 mm, 3 µm particle size, 100 Å pore size (Phenomenex, Torrance, CA, USA) maintained at 40 °C. The solvents used were A (water:acetonitrile 95:5, *v*/*v*) and B (water:acetonitrile 5:95, *v*/*v*); both acidified with 0.1% (*v*/*v*) formic acid. 

For validation of high-resolution *S. aureus* growth inhibition profiling, a test mixture consisting of metronidazole, myrcetin, podophyllotoxin, ampicillin, and tetracycline were mixed in methanol at a concentration of 60 μg/mL in methanol. Furthermore, pure ciprofloxacin (0.125 mg/mL in MeOH), the crude extract of *Alternaria* sp. (25 mg/mL in MeOH) as well as the crude extract of *Alternaria* sp. spiked with ciprofloxacin (to a ciprofloxacin concentration of 0.125 mg/mL), were also prepared. The four samples were separated (injection volume of 10 μL) using the following elution gradient profile: 0 min, 0% B; 30 min, 70% B; 33 min, 100% B, 38 min, 100% B, 39 min, 0% B, 42 min, 0% B. The eluate from of the test mixture (from 9 to 21 min) and the two extracts (from 8 to 28 min) was fractionated into 88 wells of 96-well microplates, yielding a resolution of 7.3 and 4.4 data points per min, respectively. Subsequently, the microplates were evaporated to dryness using an SPD121P Savant SpeedVac concentrator equipped with an OFP400 oil Free Pump and an RVT400 Refrigerated Vapor Trap (Thermo Scientific, Waltham, MA, USA). The *S. aureus* growth inhibition assay was performed as described above.

### 3.12. Analyses by HPLC-PDA-HRMS 

HPLC-PDA-HRMS analyses were performed on an Agilent 1200 HPLC coupled to a micrOTOF-Q II mass spectrometer (Bruker Daltonik, Bremen, Germany) equipped with an electrospray ionization source. Mass spectra were acquired in both positive- and negative-ion mode at 200 °C drying temperature, a capillary voltage of 4100 V and 4000 V, respectively, a nebulizer pressure of 2.0 bar, and a drying gas flow of 7 L/min. A solution of sodium formate clusters was automatically injected at the beginning of each run to enable internal mass calibration. Chromatographic separation was obtained using the same conditions as described for the high resolution *S. aureus* growth inhibition profiling.

## 4. Conclusions

A dye-free broth microdilution assay was developed and validated for high-resolution bioactivity profiling of the antibacterial potential of crude natural product extracts against *S. aureus* based on OD_600_ measurements. The assay was shown to be fast, reproducible, robust against temperature fluctuations and the interference from coloured crude extracts was acceptable. Based on findings of an IC_50_ value of DMSO of 6% *v/v* against *S. aureus*, the assay was developed using a DMSO concentration of 5% *v*/*v*, which proved sufficient to dissolve both crude and microfractionated extracts. Through high-resolution growth inhibition profiling of both an inactive fungal extract spiked with ciprofloxacin a well as the crude extract of *A. montevidensis*, the potential to expedite future antibacterial drug discovery from nature was highlighted.

## Figures and Tables

**Table 1:** IC_50_ values (µg/mL) of crude extracts and the positive control streptomycin (spiked) given as mean values ± standard deviations of individual experiments conducted on three consecutive days.

	Streptomycin	*A. montevidensis*	*T. asperellum*	*Alternaria* sp.	*J. oxycedrus*
37 °C	0.023 ± 0.00	39.91 ± 1.36	25.94 ± 0.62	n.d.	n.d.
34 °C	0.022 ± 0.00	41.21 ± 0.56	24.98 ± 1.21	n.a.	n.a.
40 °C	0.025 ± 0.00	39.82 ± 1.39	26.29 ± 0.89	n.a.	n.a.
Spiked (37 °C)	-	-	-	0.02 ± 0.00	0.05 ± 0.00

n.d.: no inhibition observed; n.a.: not assessed.

## Data Availability

The data presented in this study are available on request from the corresponding author.

## Supplementary Materials

The following are available online: Table S1: ITS, Alt a 1, and GAPDH sequences of *Alternaria sp.* strain ILF-013, Table S2: ITS and b-tubulin sequences of *Trichoderma asperellum* strain ILF-006, Table S3: ITS and b-tubulin sequences of *Aspergillus montevidensis* strain ILF-009.

## Sample Availability

Samples of the compounds are not available from the authors.
