# Supplementary material for: Coupling Microplate-Based Antibacterial Assay with Liquid Chromatography for High-Resolution Growth Inhibition Profiling of Crude Extracts: Validation and Proof-of-Concept Study with Staphylococcus aureus
Source: Molecules. 2021 Mar 11;26(6):1550. doi: 10.3390/molecules26061550 (PMC8001363; doi:10.3390/molecules26061550)
Supplement: Supplementary file 1 [file molecules-26-01550-s001.pdf]

Supporting information for

Coupling Microplate-based Antibacterial Assay with Liquid Chromatography for High-resolution Growth Inhibition Profiling of Crude Extracts: Validation and Proof-of-Concept Study with *Staphylococcus aureus*

Hamidreza Ardalani[1,†], Syariful Anam[1,2,†], Kresten J.K. Kromphardt[3], Dan Staerk[1] and Kenneth T. Kongstad[1,*]

[1] Department of Drug Design and Pharmacology, Faculty of Health and Medical Sciences, University of Copenhagen, Universitetsparken 2, DK-2100 Copenhagen, Denmark; hamidreza.ardalani@agro.au.dk (H.A.); kenneth.kongstad@sund.ku.dk (K.T.K.); ds@sund.ku.dk (D.S.)

[2] Department of Pharmacy, Faculty of Mathematics and Sciences, Tadulako University, Jalan Soekarno Hatta Km. 9, 94118, Palu, Central Sulawesi, Indonesia; syariful.anam@untad.ac.id

[3] Department of Biotechnology and Biomedicine, Technical University of Denmark, Søltofts Plads, Building 223, 2800 Kongens Lyngby, Denmark; krjko@dtu.dk

* Correspondence: kenneth.kongstad@sund.ku.dk; Tel.: +45 35336411

† These authors contributed equally.

Table S1. ITS, Alt a 1, and GAPDH sequences of *Alternaria* sp. strain ILF-013

Table S2. ITS and β-tubulin sequences of *Trichoderma asperellum* strain ILF-006

Table S3. ITS and β-tubulin sequences of *Aspergillus montevidensis* strain ILF-009

Table S1. ITS, Alt a 1, and GAPDH sequences of *Alternaria* sp. strain ILF-013

Gene	Sequence
ITS	CGTAGGTGAACCTGCGGAGGGATCATTACACAATATGAAAGCGGGCTGGCATC<br>CTTCGGGGTTACAGCCTTGCTGAATTATTCACCCGTGTCTTTTGCGTACTTCTTG<br>TTTCCTTGGTGGGTTCGCCCACCATAGGACAAACCATAAACCTTTTGTAATTGC<br>AATCAGCGTCAGTAAAAAAATTAATAATTACAACTTTTAACAACGGATCTCTT<br>GGTTCTGGCATCGATGAAGAACGCAGCGAAATGCGATAAGTAGTGTGAATTGC<br>AGAATT<br><br>Accession Number: MT755729
Alt a 1	CTTGCCGCCGCCGCTCCCCTTGAGTCTCGCCAGGACACTGCATCCTGCCCTGTC<br>TCCACCCAGGGTGACTACGTCTGGAAGATCTCTGAGTTCTACGGACGCAAGCC<br>TGAGGGAACCTACTACAACAGCCTAGGGTTCAACATCAAGGCCACCAACGGAG<br>GAACCCTCGACTTCACCTGCTCTGCTCAGGCGGACAAGCTTGAGGACCACAAG<br>TGGTACTCTTGCGGCGAGAACAGCTTCATGGACTTTTCTTTTGACAGCGACCGC<br>AGCGGTCTGCTCCTGAAGCAGAAGGTCAGCGACGAGTAAGTTTCCCCCATACT<br>CTCGAATACTCCTCATCTTCAGATGTACTAACTTTTCCTTCAGCATCACCTATGT<br>CGCTACCACCACTCTTCCTAACTACTGCCGCGCTGGCGGTAACGGCCCCAAGG<br>ACTTCGTCTGCCAGGGCGTCTCCGACGCCTGCATCACCCTCGT<br><br>Accession Number: MT759799
GAPDH	AGCTTCACCCGCTCCATTGATTCAATTGTATCAAAGCTAACCGCATGTCACAGT<br>ATCGAGCACAACGACGTCGACATTGTCGCCGTAAACGACCCCTTCATCGAGCC<br>CCACTACGCTGTAAGCTTCCCCAAGCACTCAAACTATAGCCATAGCCATCCAA<br>ATCGCGACATCAGTCCTTGCGATGCGCTAGGGCTACTCCACGGTTGCAGATTGC<br>AGGCTAACACATCCAGGCCTACATGCTCAAGTATGACAGCACACACGGCCAGT<br>TCAAGGGCGAGATCAAGGTTGACGGCAACAACCTGACCGTCAACGGCAAGAC<br>CATCCGTTTCCACATGGAGAAGGACCCCGCCAACATCCCATGGAGCGAGACCG<br>GCGCTTACTACGTCGTTGAGTCCACCGGTGTCTTCACCACTACCGAGAAGGCCA<br>AGGCTCACTTGAAGGGTGGAGCCAAGAAGGTCGTCATCTCTGCTCCCTCTGCT<br>GACGCCCCCATGTTCGTCATGGGTGTCAACCACGAGACTTACAAGTCCGACAT<br>TGAGGTTCTCTCAAACGCCTCTTGCACAA<br><br>Accession Number: MT759800

Table S2. ITS and β-tubulin sequences of *Trichoderma asperellum* strain ILF-006

Gene	Sequence
ITS	AACAAGGTCTCCGTTGGTGAACCAGCGGAGGGATCATTACCGAGTTTACAACT<br>CCCAAACCCAATGTGAACGTTACCAAACTGTTGCCTCGGCGGGGTCACGCCCC<br>GGGTGCGTCGCAGCCCCGGAACCAGGCGCCCGCCGGAGGAACCAACCAAACT<br>CTTTCTGTAGTCCCCTCGCGGACGTATTTCTTTACAGCTCTGAGCAAAAATTCA<br>AAATGAATCAAAACTTTCAACAACGGATCTCTTGGTTCTGGCATCGATGAAGA<br>ACGCAGCGAAATGCGATAAGTAATGTGAATTGCAGAATTCAGTGAATCATCGA<br>ATCTTTGAACGCACATTGCGCCCGCCAGTATTCTGGCGGGCATGCCTGTCCGAG<br>CGTCATTTCAACCCTCGAACCCCTCCGGGGGATCGGCGTTGGGGATCGGGACC<br>CCTCACACGGGTGCCGGCCCCTAAATACAGTGGCGGTCTCGCCGCAGCCTCTC<br>CTGCGCAGTAGTTTGCACAACTCGCACCGGGAGCGCGGCGCGTCCACGTCCGT

	AAAACACCCAACTTTCTGAAATGTTGACCTCGGATCAGGTAGGAATACCCGCT<br>GAACTTAAG<br><br>Accession Number: MT755730
β-tubulin	GGTGCTGCTTTCTGGCAAACCATTTCCGGCGAGCACGGCCTCGACAGCAATGG<br>TATCTACAACGGCTCTTCTGAGCTCCAGCTGGAGCGCATGAACGTCTACTTCAA<br>CGAGGTATGTCTGGCTACGCCAAAAGAGGACACCTGGAAGACGTCCCGTTCTG<br>ACCTTGATGGTGCAGGCCTCCAACAACAAGTATGTTCCTCGCGCTGTCCTCGTC<br>GATCTCGAGCCCGGCACCATGGACGCCGTCCGTGCCGGTCCTTTCGGTCAGCTC<br>TTCCGTCCCGACAACTTCATCTTCGGCCAGTCCAGTGCCGGAAACAACTGG<br><br>Accession Number: MT759801

Table S3. ITS and β-tubulin sequences of *Aspergillus montevidensis* strain ILF-009

Gene	Sequence
ITS	AACAAGGTTTCCGTAGGTGAACCTGCGGAAGGATCATTACCGAGTGCGGGCCC<br>TCTGGGTCCAACCTCCCATCCGTGTCTATCTGTACCCTGTTGCTTCGGCGTGGC<br>CACGGCCCGCCGGAGACTAACATTTGAACGCTGTCTGAAGTTTGCAGTCTGAG<br>TTTTTAGTTAAACAATCGTTAAAACTTTCAACAACGGATCTCTTGGTTCCGGCA<br>TCGATGAAGAACGCAGCGAAATGCGATAATTAATGTGAATTGCAGAATTCAGT<br>GAATCATCGAGTCTTTGAACGCACATTGCGCCCCCTGGTATTCCGGGGGGCAT<br>GCCTGTCCGAGCGTCATTGCTGCCCTCAAGCACGGCTTGTGTGTTGGGCTTCCG<br>TCCCTGGCAACGGGGACGGGCCCAAAAGGCAGTGGCGGCACCATGTCTGGTCC<br>TCGAGCGTATGGGGCTTTGTCACCCGCTCCCGTAGGTCCAGCTGGCAGCTAGCC<br>TCGCAACCAATCTTTTTAACCAGGTTGACCTCGGATCAGGTAGGGATACCCGCT<br>GAACTTAAGCATAT<br><br>Accession Number: MT755731
β-tubulin	GGTGCTGCTTTCTGGTATGTTTTTAAAATATTGGGAGATGGATTGGGAGATATA<br>CTAACAGTATCACAGGCAGACTATCTCCGGCGAGCACGGTCTCGACGGCTCTG<br>GTGTGTAAGTACAGTCGGGTCTCCGAGATGGACGCGTATCGGATATGGATATC<br>TAACGGATTTCAGCTACAACGGATCCTCCGACCTCCAGTTGGAGCGTATGAAC<br>GTCTACTTCAACGAGGTTTGCCTATCCGTTCGTGTTTGTGTGGAAACAGCTCTG<br>ACAGTGATAGGCCTCCAACAACAAATATGTCCCCCGTGCCGTCCTCGTCGACCT<br>TGAGCCAGGTACCATGGATGCCGTCCGTGCCGGTCCCTTCGGTCAGCTCTTCCG<br>CCCCGACAACTTCGTCTTCGGCCAGTCCGGTGCCGGTAACAACTGGGCCAAGG<br>GTC<br><br>Accession Number: MT759802
